# Correlation between positron annihilation lifetime and photoluminescence measurements for calcined Hydroxyapatite

**DOI:** 10.1038/s41598-024-59855-1

**Published:** 2024-05-06

**Authors:** Hoda Atta, Kamal R. Mahmoud, Elsayed I. Salim, Eithar Elmohsnawy, Abdelhamid El-Shaer

**Affiliations:** 1 Physics Department, Faculty of Science, Kafrelsheikh University, Kafrelsheikh, 33516 Egypt; 2 Research Lab. of Molecular Carcinogenesis, Zoology Department, Faculty of Science, Tanta University, Tanta, 31527 Egypt; 3 Department of Botany, Faculty of Science, Kafrelsheikh University, Kafrelsheikh, 33516 Egypt

**Keywords:** Hydroxyapatite, Zeta potential, Positron annihilation, Photoluminescence, Biophysics, Materials science, Nanoscience and technology, Physics

## Abstract

Hydroxyapatite (HAp) Ca_10_(PO_4_)_6_(OH)_2_ is a compound that has stable chemical properties, composition, and an affinity for human bone. As a result, it can be used in odontology, cancer treatment, and orthopedic grafts to repair damaged bone. To produce calcined HAp at 600 °C with different pH values, a wet chemical precipitation method was employed. All synthesized HAp samples were characterized by X-ray diffraction (XRD), Raman spectroscopy, scanning electron microscopy (SEM), transmission electron microscopy (TEM), Fourier-transform infrared spectroscopy (FTIR), photoluminescence (PL), Zeta potential, and positron annihilation lifetime spectroscopy (PALS). The XRD results revealed that all calcined HAp samples were formed in a hexagonal structure with a preferred (002) orientation at different pH values. The crystal size of the samples was determined using the Scherrer equation, which ranged from 16 to 25 nm. The SEM and TEM results showed that the morphology of the samples varied from nanorods to nanospheres and rice-like structures depending on the pH value of the sample. The PL measurements indicated that the blue and green emission peaks of HAp were due to defects (bulk, surface, and interface) in the samples, which created additional energy levels within the band gap. According to Zeta potential measurements, the charge carrier changed from a positive to negative value, ranging from 3.94 mV to − 2.95 mV. PALS was used to understand the relationship between the defects and the photoluminescence (PL) properties of HAp. Our results suggest that HAp nanoparticles have excellent potential for developing non-toxic biomedical and optical devices for phototherapy.

## Introduction

Hydroxyapatite or HAp [Ca_10_(Po_4_)_6_(OH)_2_] is a substance commonly found in nature with remarkable bioactive properties. It is ideal for bone implants due to its excellent osteoconductive, biocompatibility, bioactivity, and chemical properties, all of which closely resemble the mineral components of human bones^1^. The structure of HAp consists of phosphate clusters ([PO_4_]) that form a hexagonal crystal structure called space group P6_3_/m^2^. The composition of HAp is Ca(1)4Ca(2)6(PO_4_)6(OH)_2_, with different Ca species occupying different crystallographic locations. The Ca (1) sites are surrounded by nine O atoms from PO_4_ tetrahedral, which create [CaO_9_] clusters due to the structure's ternary orientation. The hexagonal channels of the structure have Ca (2) sites at their corners, which are surrounded by [PO_4_] clusters made up of six O atoms, and an OH group that produces [CaO_7_H] clusters within the channel. Even with vacancies and various ionic substitutions, the HAp lattice can maintain its hexagonal shape^3,4^. HAp exhibits poor mechanical properties, which limits its use in orthopedic applications that require load-bearing capabilities. However, its properties can be improved by modifying the synthesis conditions and doping the material with other elements^5^. It is also possible to convert it into a magnetic nanocomposite, which has potential uses in biomedical fields^6^. Doping HAp with transition metal ions such as chromium can enhance its electrical properties^7^. Furthermore, the optical properties of HAp can vary depending on several factors, including synthesis conditions^8^. Therefore, HAp is a versatile material with a wide range of properties that can be tailored to specific medical applications.

There are multiple methods available for synthesizing Hydroxyapatite (HAp), including solid-state synthesis, hydrothermal synthesis, sol–gel method, and wet chemical precipitation method^9–11^. Among these methods, wet chemical precipitation is a useful technique that can be utilized to produce different particle forms and create various structural imperfections. This can help in understanding its Photoluminescence effect^12^.

Although fluorescent compounds are highly favored for use in medical applications such as bioimaging and drug delivery due to their intense and controllable emissions, developing novel compounds with efficient and customizable optical characteristics has become an obstacle for the industry. However, previous investigations have yielded inorganic components with luminescence centers^13,14^. One such system is the HAp system, which can be used as fluorescent agents for bioimaging and biolabeling, monitored drug delivery systems, and white-light emitting diodes^15–21^. HAp was previously employed as a host for these luminescence centers, especially lanthanides. Annemarie et al. suggested biocompatible Eu^3+^ and Dy^3+^ doped hydroxyapatite for luminescence imaging, which can serve as a contrast agent for MRI in permanent implants or functional coatings^22^. Meanwhile, Neacsu et al. focused on doping HAp with other rare biomedical elements such as terbium (Tb^3+^), erbium (Er^3+^), europium (Eu^3+^), lanthanum (La^3+^), and dysprosium (Dy^3+^)^23^. Sudip et al. proposed doping HAp with rare earth ions Gadolinium and europium (Gd^3+^, Eu^3+^) to enhance luminescence for biological fluorescence imaging applications^24^. Recently, Kolesnikovs et al. studied HAp doping with Ce^3+^ for luminescence applications^25^. Although HAp samples are expected to exhibit intense self-activated photoluminescence (PL) due to their high density of structural and surface defects caused by chemical synthesis at low temperatures^2,26,27^.

So, our goal was to produce pure hydroxyapatite without using any capping agents, using a simple low-temperature method that relied on adjusting the pH value and annealing the material to improve its intense self-activated photoluminescence (PL). The PL effect was due to the high density of structural and surface defects in the material, which we analyzed using positron annihilation lifetime spectroscopy (PALS), a precise and accurate technique. We then explained the relationship between the positron annihilation lifetime and photoluminescence measurements.

### Materials and methodology

**Figure 1:**
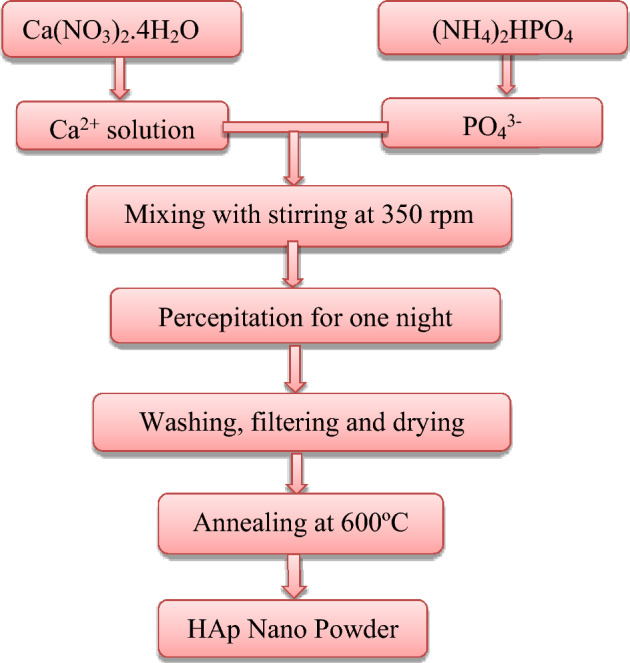
Schematic diagram shows the hydroxyapatite preparation by using chemical percepitation method.

The wet chemical precipitation method was used to create HAp samples at room temperature with varying pH levels. As shown in Fig. 1, the starting precursors were calcium nitrate tetrahydrate [Ca(NO_3_)_2_·4H_2_O] (99%, Sigma Aldrich) and di-ammonium hydrogen phosphate [(NH_4_)2HPO_4_] (98%, Sigma Aldrich), with the pH of the solutions adjusted by adding ammonium hydroxide [NH_4_OH] (Junsei Chem.Ltd., Japan). Deionized water was used as a solvent. Two groups were synthesized through chemical precipitation—one in an acidic environment without the addition of NH_4_OH (final pH = 5), and the other in a basic environment with the addition of NH_4_OH (final pH = 7, 9 and 11). Additionally, to enhance their properties, the samples were annealed at 600 ºC. The chemical solution used in this experiment consisted of two substances: 0.1 M Ca(NO_3_)_2_·4H_2_O and 0.06 M of [(NH_4_)_2_HPO_4_]^28,29^. The precursors were dissolved in separate beakers containing 100 ml of deionized water. The Calcium nitrate tetrahydrate [Ca(NO_3_)_2_·4H_2_O] solution was added at a constant rate of 3ml/min to the phosphate solution and mixed completely at 30 ºC by stirring with a magnetic stirrer at 350 rpm. Ammonium hydroxide was used as a complex agent to adjust the pH value to different levels (7, 9 and 11). During the synthesis, the solution eventually turned milky white, and the reaction was completed with the formation of a thick white precipitate. To eliminate any contaminants, the precipitate was washed with distilled water and ethanol after being aged overnight. The samples were then weighed and subjected to structural and optical characterization. The reaction can be expressed using the equation below:

1 $$10{\text{Ca}}{\left({{\text{NO}}}_{3}\right)}_{2}4{{\text{H}}}_{2}{\text{O}}+6{\left({{\text{NH}}}_{4}\right)}_{2}{{\text{HPO}}}_{4}+8{{\text{NH}}}_{4}{\text{OH}}\to {{\text{Ca}}}_{10}{({{\text{PO}}}_{4})}_{6}{({\text{OH}})}_{2}+20{{\text{NH}}}_{4}{{\text{NO}}}_{3}+10{{\text{H}}}_{2}{\text{O}}$$

X-ray diffraction and scanning electron microscopy were used to study the structural and morphological properties of the powder, which was fabricated using the Shimadzu (plate number 1) at Kafrelsheikh University, along with the JSM-IT 100 microscope. Photoluminescence spectroscopy was used to measure the optical properties of the samples. The PL was studied using a 3.82 eV (325 nm) Kimmon He-Cd laser, and the spectra were recorded on a HORIBA iHR320 spectrometer, using the built-in Synapse CCD camera. The Raman spectra were demonstrated using a WITec alpha300 R system. The positron lifetime measurements were carried out using a fast–fast coincidence ORTEC system, with a 20 mCi 22NaCl source. The 22NaCl solution was deposited on Kapton foil and sandwiched between Hap nanoparticle samples. The spectra were analyzed using the LT computer program. The Zeta Potential Analyzer Ver.5.59 was utilized for zeta potential analysis, with samples dissolved in Milli-Q water at a concentration of 0.30 mg ml-1, using a 10kJ tip ultrasonic.

## Results and analysis

### XRD results

**Figure 2:**
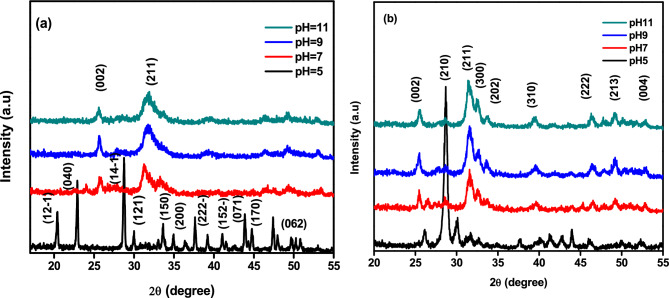
XRD data for HAp samples at various pH (**a**) before annealing and (**b**) after annealing.

Figures 2a and b display the XRD pattern for the samples prepared by the wet precipitation method. These samples were synthesized with various pH values, and Fig. 2a shows the pattern before annealing while Fig. 2b shows the pattern after annealing. The synthesized samples at pH 5 exhibited brushite as the predominant phase, with card no. (JCPDS 01-072-0713). Additionally, some peaks of hydroxyapatite with card no. (JCPDS 09-0432) were also observed. The miller indices for these peaks were (12-1), (040), (14-1), (121), (150), (15-2), (170), and (062). It is worth noting that the brushite formation was kinetically favored in acidic pH levels^30^. The brushite phase exhibits narrow diffraction peaks at half maximum intensity, indicating strong crystallinity. In contrast, the hydroxyapatite phase displays broad peaks that appear upon increasing the pH value to 7, 9, and 11, respectively, in the same figure. The XRD pattern of annealed samples at 600ºC is shown in Fig. 2b. This pattern displayed high crystallinity, as evidenced by the narrow and sharp diffraction peaks. In comparison, Fig. 2a showed broader diffraction peaks. At the lowest pH level, heat treatment transformed the synthesized brushite to calcium pyrophosphate (JCPDS 09-0346). Recent investigations have suggested that calcium pyrophosphate and β-TCP could be a viable alternative to pure hydroxyapatite in clinical settings^31,32^. The main diffraction peaks of hydroxyapatite (JCPDS 09-0432) were observed in all samples at physiological and high pH levels with a preferred orientation (002), (211), (300), (202), (310), (222), (213), and (004) planes positioned at 25.59°, 28.355°, 31.75°, 32.845°, 34.024°, 39.812°, 46.527°, 49.249°, and 52.944°, respectively, by raising the pH value to 7, 9, and 11. The XRD pattern revealed that the as-prepared HAp had a poor crystalline phase, which was improved by annealing, resulting in a single calcium phosphate phase at pH 11. The samples of our hydroxyapatite (HAp) displayed diffraction patterns that suggest either nanometric crystallite size or poor crystallinity, like in vivo bone tissue created in physiological and higher pH values^30^. The diffraction peaks could be accurately assigned to the pure hexagonal HAp phase (space group P6_3_/m), and the average lattice parameter of the prepared and calcined samples were calculated to be almost equal to the standard values, with a = b = 9.41 Å, and c = 6.91 Å, α = β = 90, and γ = 120. We used Scherrer's equation^34,35^ to determine the average crystallite size.

2 $${\text{D}}=\frac{\mathrm{K\lambda }}{\beta cos\theta }$$

The average size of crystals in nanometers is represented by D, while K is the constant value of the shape factor, which is fixed at 0.9. λ denotes the wavelength of radiation, β represents the full width at half-maximum (FWHM), and θ is the diffraction angle. Table 1 displays the measured average crystal size for both the as-prepared samples and after annealing.

**Table 1:** The average crystal size for HAp samples at various pH, before annealing and after annealing.

Sample	As-prepared group	Annealed group
pH5	22.3 nm	25.23 nm
pH7	13.85 nm	16.48 nm
pH9	13.69 nm	16.46 nm
pH11	13.28 nm	16.3 nm

According to measurements, the particle size decreased as the pH values increased^36^. However, the particle size increased after annealing due to agglomeration caused by the heat treatment^37^. Therefore, the synthesis conditions heavily influence the production of hydroxyapatite (HAp) as a single phase or in conjunction with other orthophosphate phases^38^.

## Morphological features

**Figure 3:**
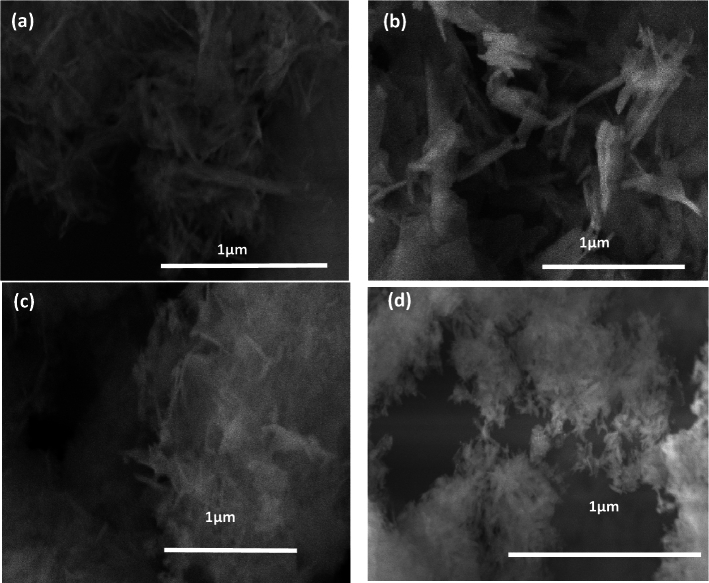
FE-SEM for calcined HAp samples at various pH (**a**) 5, (**b**) 7, (**c**) 9 and (**d**) 11.

Figure 3a–d display the FE-SEM images taken to examine the morphology of the prepared samples with various pH values that were annealed at 600 °C. Figure 3a depicts the FE-SEM micrograph of the sample that was prepared in an acidic environment without the addition of NH_4_OH (pH5). It appears to have a nanorod shape. Increasing the pH value led to a reduction in particle size and changes in shape, which is supported by the XRD results. The shapes of the crystals changed from nano rods to nano wires, and agglomeration of nano rice-like structures. It is known that the initial pH levels affect the ion balance in the solution, which in turn influences the concentration of OH^−^, Ca^2+^, PO_4_^3−^, and (HPO_4_)^2−^. In Fig. 3b, for higher pH values equal to 7, the presence of the hydroxyl group in the solution during the growth of the Hap crystal affects the form of the crystal's planes a, b, and its growth direction (C axis). In Fig. 3c, the sample with a pH of 9.00 showed slow OH-Ca_6_ formation on the a and b axes, but Ca^2+^ was sufficient on the c axis to generate Ca-P_6_O_24_, resulting in HAp nanowires^39^. In Fig. 3d, it can be observed that increasing the pH level to 11 inhibits the formation of HAp, causing it to precipitate or form solid phases. This reduces its availability for chemical bonding due to the changes in solution equilibrium. At pH 11, the form of HAp changes to agglomerated rice grain-like structures because of the high concentration of hydroxide ions, which prevent extension^28,40^.

### Transmission electron microscope (TEM) results

**Figure 4:**
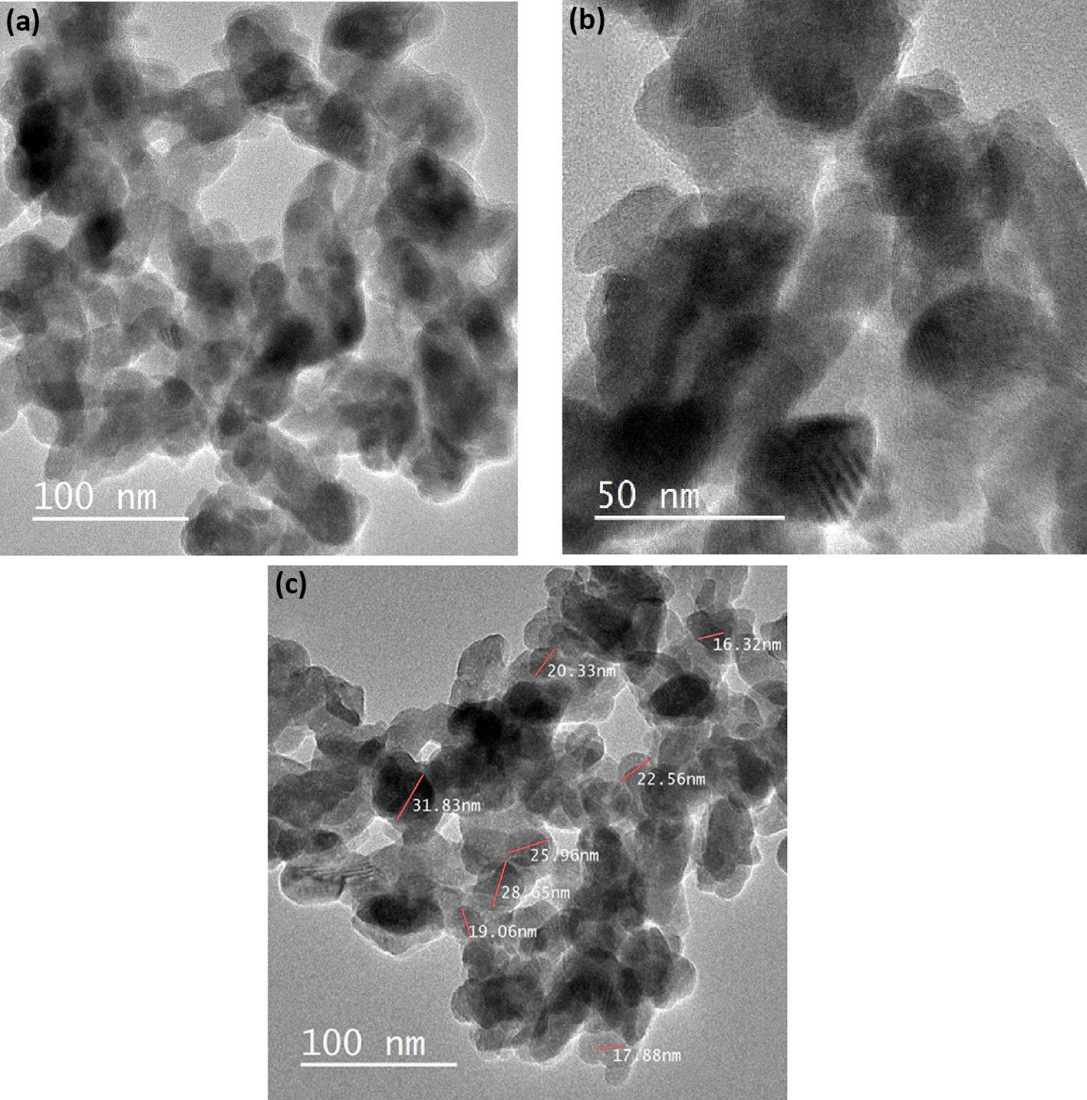
TEM image for calcined HAp sample at pH 11 (**a**) agglomeration of spherical and nano rice shape at 100nm (**b**) at 50nm (**c**) size particle distribution of the sample.

Figure 4a–c show TEM images of hydroxyapatite sample annealed at 600ºC with a pH of 11. As seen in Fig. 4a and b the sample consists of agglomerations of spherical and rice-like nanostructures, caused by the high concentration of hydroxide ions (OH^-^) at high pH values. This led to a rapid growth of the a, b, and c axes at the same speed due to the rapid attraction between the negative charge of the HAp nucleus and the positive charge of Ca-OH_6_^39,41^. As a result of increased solubility, inhibition occurred, causing changes in the chemical equilibrium. This was due to the abundance of hydroxide ions (OH^-^) which greatly influenced the growth of primary unit particles and led to saturation of the solution at high pH values^42–44^. In Fig. 4c, the particle size distribution is shown to range from 16 to 30 nm, with an average particle size of 22.8 nm, highlighting its fabrication in nano size.

## Raman spectroscopy results

**Figure 5:**
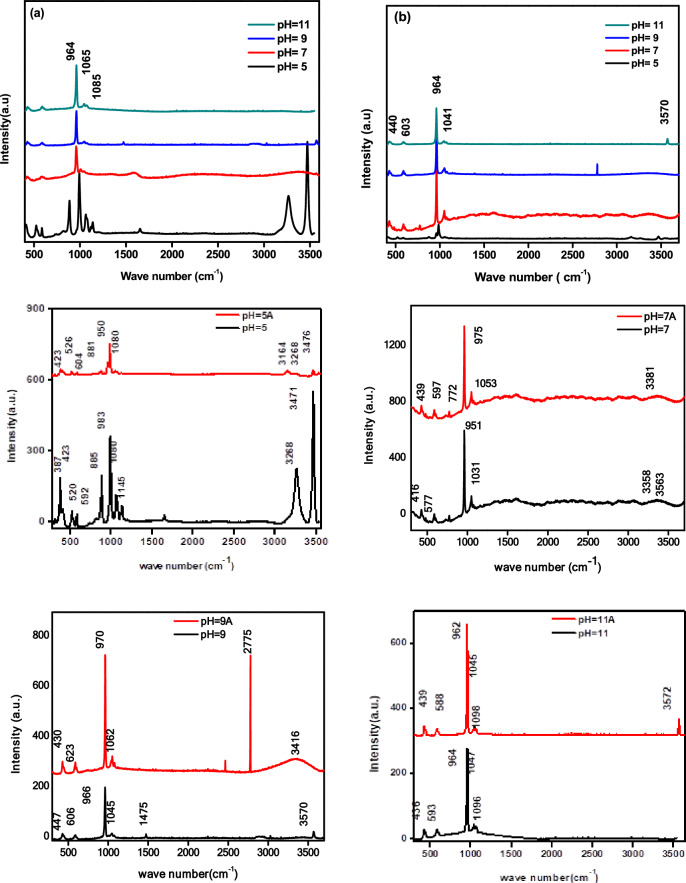
Shows the Raman spectrum of HAp (a) before annealing and (b) after annealing.

Figure 5a and b show the Raman spectra of prepared samples that were created with different pH levels before and after annealing. In Fig. 5a, the Raman spectroscopy of brushite crystals (CaHPO_4_.2H_2_O) synthesized using wet chemical precipitation at pH 5 exhibited a distinct peak at 885 cm^-1^, which corresponds to the phosphate group (P-O(H)). This is different from the Raman spectra of other samples^45^. On the other hand, the Raman spectrum of HAp at pH 7, 9, and 11 reveals a characteristic tetragonal PO_4_^3−^ (*v*_1_) group peak at 987 cm^−1^, the PO_4_^3−^ (*ν*_2_) vibrational mod O−P−O bending modes at 430 cm^−1^. The bands at 1065, 1085, and 1151 cm^−1^ are due to the asymmetric stretching of P−O(*ν*_*3*_), which indicates the existence of a disordered phosphate lattice in apatite. The phases of Brushite and HAp have different Raman spectra, showing distinct shifts in PO_4_^3−^. These shifts appear at 413 and 587 cm^-1^ and represent the symmetric bending mode's *ν*_*2*_ and *ν*_*4*_. Additionally, the hydrogen phosphate anions found in synthetic brushite have vibrational modes and bands at 526 and 542 cm^−1^. The stretching mode of the water molecule is indicated by the bands at 3276 and 3471 cm^−1^^46^. The HPO_4_^2-^ molecule's antisymmetric stretching mode (*ν*_*3*_) is represented by the bands at 1118 and 1137 cm^-1^. In Fig. 5b, the synthesis of pure Hap was completed at pHs 7, 9, and 11. The functional bands of (PO_4_)^3-^, hydroxyl (OH^-^) appeared at 463, 593, and 606 cm^−1^, which correlates to the *ν*_*2*_ and *ν*_*4*_ O–P–O. The bands that appeared at 966, 1045, and 1096 cm^−1^ correspond to *v*_*1*_ symmetric and *v*_*3*_ asymmetric stretching modes of P-O^47,48^. It has been found that certain organic materials exhibit bands at 1555 and 1650 cm^−1^, which can be eliminated through the process of annealing^49,50^. Through annealing, all HAp samples bands were detected, which became more pronounced and narrower with increasing temperature. This indicates that the presence of phosphate ions increases, and HAp crystallization occurs at higher temperatures. The hydroxyl group's symmetric, stretching mode can be observed at 3570 and 629 cm^−1^. The peaks of the phosphate ions (PO_4_)^3−^ accurately appear between 961 and 964 cm^−1^, which is the most intense peak. The overlapping of bands around 1045–1049 cm^−1^ is attributed to the phosphate vibrations^50,51^. These vibrations involve the symmetric, stretching mode of the phosphate group, indicating the presence of the hydroxyapatite crystalline structure. The bands of carbonate ions at 1475 cm^−1^ could be due to atmospheric adsorption^52,53^.

## FTIR results

**Figure 6:**
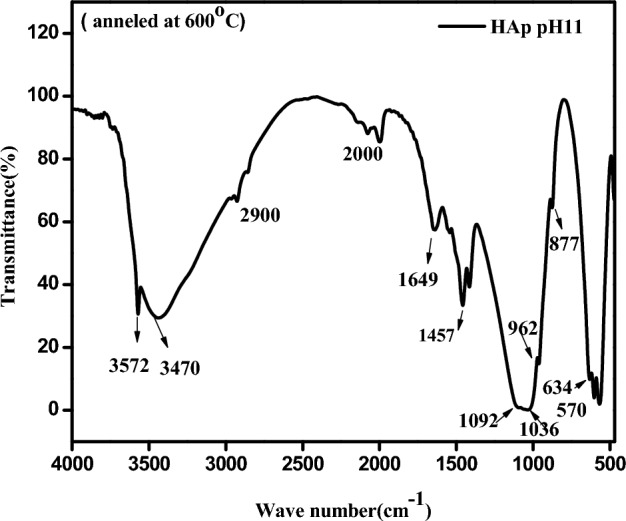
FTIR spectrum of HAp sample pH11 annealed at 600°C.

Figure 6 depicts the FTIR spectra of the HAp sample synthesized with pH11 and annealed at 600ºC. The adsorption bands that appeared between 570–610, 962–1052, and 1092cm^−1^ signify the symmetric bending, symmetric stretching, and asymmetric stretching vibrations of the phosphate (PO_4_)^3−^ groups, respectively. The peak at 877 cm^−1^ represents the P-O bending vibration for (PO_4_)^3−^ group, as stated in^28^. Additionally, there are three more peaks at 1410, 1457 and 1649 cm^−1^ which represent the stretching vibrations of C–O, C–O and C=O of the carbonate ions respectively. The minor peaks centered at 2000 and 2900 cm^−1^ show the triple bond C≡C and the C–H stretching vibrations of impurities found in the sample^54^.

. The peaks observed at 630, 3443, and 3572 cm^-1^ indicate the bending and stretching vibrations of the hydroxyl group (OH)^−^^28^. These findings authenticate the existence of hydroxyapatite fingerprint patterns, which confirms the presence of the hydroxyapatite phase in the synthesized samples at pH11 after annealing. These results agree with the Raman results.

### Optical characterization

#### Photoluminescence spectroscopy

**Figure 7:**
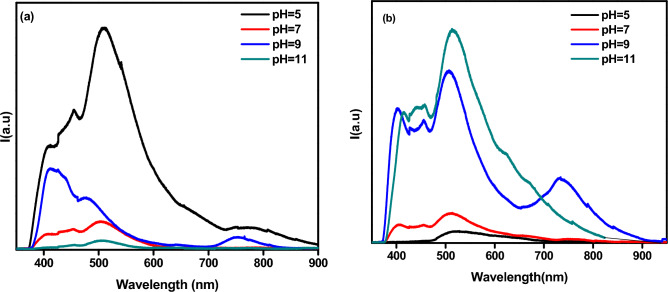
PL Spectrum of HAp (**a**) before annealing (**b**) after annealing.

In this study, the photoluminescence spectra of calcium hydroxyapatite samples were observed at different pH levels (5, 7, 9, and 11) before and after annealing at 600 °C as shown in Fig. 7a and b. The highest photoluminescence peak was observed at around 400 and 515 nm. The sample analysis in Fig. 7a revealed that the photoluminescence intensity decreased with an increase in pH value (7, 9, and 11) in. This decrease could be attributed to the lower concentration of impurity luminescence centers (CO_3_^2−^) in the samples. This finding is supported by previous studies^26,55^. After annealing, the luminescence behavior of identical samples was reversed, as shown in Fig. 7b. This result is attributed to the effect of pH value, as the spherical morphology enhances luminescence and brightness due to its high backing density and reduced light scattering^56^.

Additionally, the presence of nearby OH^−^ groups can increase the number of interstitial vacancies. The decomposition of (CO_3_)^2−^ and its substitutes can also create new energy levels within the forbidden zone^2,26^. These levels, which may be either deep or shallow, are caused by surface and bulk defects at the interface^2,16^. This leads to broadening in the PL spectra during annealing. Therefore, heat treatment can alter the order and disorder of the structure, resulting in an abundance of vacancies^26^.

#### Positron annihilation spectroscopy results

**Figure 8:**
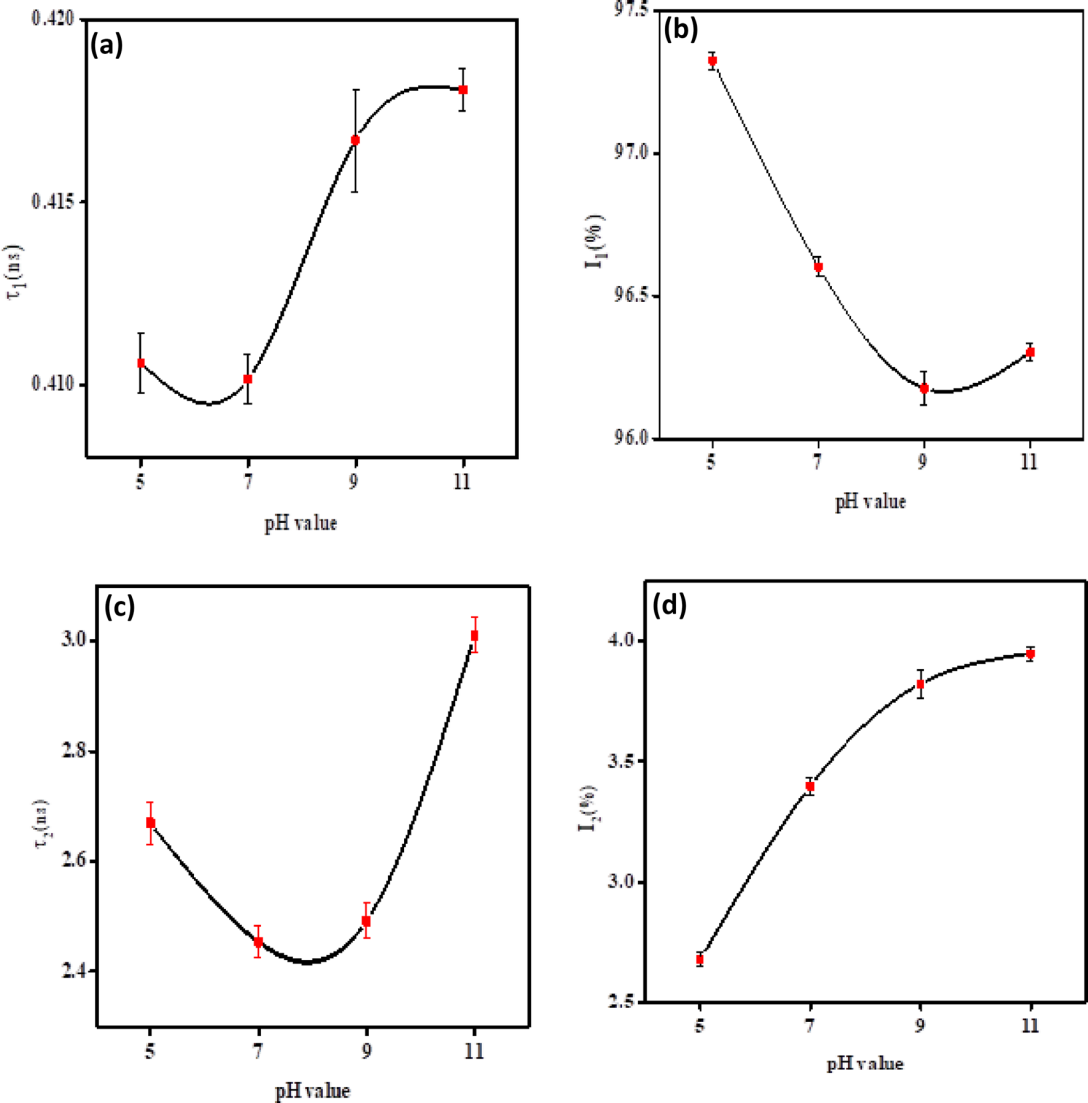
The variation of PAL components as a function of pH value of the measured samples: (**a**) τ_1_ ns; (**b**) I_1_%; (**c**) τ_2_ ns and (**d**) I_2_%).

The positron lifetime spectra of the annealed hydroxyapatite samples can be separated into two components: an intermediate-lived component (τ_1_) and a long-lived component (τ_2_) with intensities I_1_ and I_2_, respectively^57,58^. The τ_1_ component is attributed to the annihilation of free positrons and para-positronium (p-Ps), while the τ_2_ component is attributed to the annihilation of ortho-positronium (o-Ps) pick-off annihilation, which depends on the size of the free volume defect. Figure 8a and b illustrate how τ_1_ and I_1_ change with varying pH values. The range of τ1 varies from (0.4106 ± 0.00083) to (0.41808 ± 0.00057), while I1 ranges from (97.325 ± 0.031) to (96.303 ± 0.030). For pH values of 5 and 7, τ_1_ remains almost constant. However, when the pH value is increased to 11, τ_1_ increases to 0.418 ns. This increase is likely due to the higher concentration of hydroxyl groups, which increase hydrogen bonding and enhance crosslinking. As a result, the electron density decreases and τ_1_ increases while I_1_ decreases. The decrease in I_1_ may be a result of defect agglomeration, which increases the size of defects and decreases their concentration^59–62^. In Fig. 8c and d, you can see how τ_2_ and I_2_ change with varying pH values. The results indicate that τ_2_ ranges from 2.67 to 3.01 ns, while I_2_ ranges from 2.7 to 3.9%. For samples with pH values of 5 and 7, τ_2_ decreased from 2.66 to 2.45ns, which may be due to incomplete synthesis and impurities of HAp^39,55^. When the pH level was increased to 11, τ_2_ (tau_2_) increased from 2.49 to 3.01 ns. This may have been caused by the formation of larger free volumes resulting from the aggregation of smaller free volumes, which grew due to the increase in pH level^63^. It has been observed that the values of I_2_ have increased for all the samples of different pH levels, as shown in Fig. 8d. This increase in I_2_ can be attributed to several reasons, one of which could be the strong oxidation caused by the heat treatment^64,65^. It is suggested that O_2_^-^ radicals must move within the apatitic structure, causing the vacancy site to migrate in the crystalline material. This behavior is similar to that of TiO_2_, as reported by Okamoto et al. It is assumed that changes in the apatitic structure due to the removal of structural water at 900-1150ºC are influenced by the movement of holes^66,67^.

It is possible for I_2_ to increase due to heat treatment, which removes water and results in the formation of an oxo bond with a lower electron density than the hydrogen bond^68,69^. Conversely, annealing can lead to the creation of hydroxyl ion vacancies (V_OH_) due to the large number of OH^-^ groups, which causes the strength of the signal to increase as the concentration of V_OH_ grows^70^. It is also possible that a small amount of hydroxyl group may still be present even after heat treatment. This can act as an effective cation scavenger, boosting the synthesis of positronium by scavenging holes when introduced^71^.

### Zeta potential results

**Figure 9:**
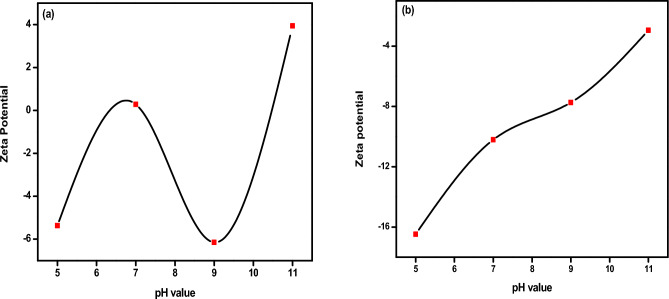
Zeta Potential Spectrum of HAp as a function of pH value (**a**) before annealing (**b**) after annealing.

Figure 9a and b display the ZP data for samples prepared with different pH values before and after annealing at 600 °C. In Fig. 9a, before annealing, the surface charge of the pH5 sample was negative (− 5.37 mv). However, as the pH value was increased, the charge carrier reversed and became positive, indicating that the sample underwent a hydrolysis process. This is because phosphate ions undergo the highest hydrolysis at low pH values^72–74^. The conversion of (PO_4_)^3-^ into HPO_4_^2−^ and H_2_PO_4_^−^ is the probable source of the negative charge. This is supported by both Fig. 9a and Table 2. Increasing the pH value beyond 5 results in a positive surface charge. This is due to the high concentration of calcium ions. After annealing, the surface charge values, In Fig. 9b were all found to be negative. This is believed to be due to the presence of OH^-^ groups, which ionize to O_2_^−^ and H^+^^75^. Additionally, both PO_4_^3−^ and OH^−^ groups are oriented on the outer surface of the Hap unit cell^76^. It's possible that particle morphology changes^77^, the presence of carbonate groups^78^, oxygen vacancies, and reversed hydrolysis could cause dehydration of the sample, resulting in the negative surface charge. By increasing the pH value, the negative surface charge of the particle decreases^79^. This decrease is believed to be caused by the dissociation of a functional group or the differential adsorption of ions from the solution on the particle's surface^80^. The sample with pH11 had the lowest negative ZP value (− 2.95 mV). This corresponds to a decrease in surface charge due to a high density of defects, which are responsible for emitting light signals and broadening the PL spectrum. These findings were confirmed by PALS, PL, and ZP measurements. Negative ZPs are beneficial in biological applications as they aid in Ca^2+^ ion adsorption, promoting extracellular matrix for cell adhesion. In contrast, nanoparticles with positive surface charges are considered more toxic^81^.

**Table 2:** Zeta potential values for HAp samples with different pH values before and after calcination.

Sample name	Zeta value before annealing	Zeta value after annealing
pH = 5	− 5.37 mV	− 16.48 mV
pH = 7	0.28 mV	− 10.21 mV
pH = 9	− 6.15 mV	− 7.74 mV
pH = 11	3.94 mV	− 2.95 mV

## Conclusion

HAp nanoparticles were successfully produced through the wet chemical precipitation method by annealing at 600°C at various pH levels. The crystal structure of the HAp nanoparticles was hexagonal, with a preferred orientation of (002) and (211), and a crystal size ranging from 16.3 to 25.23 nm. The shape of the nanoparticles varied with different pH values, ranging from nanorods to nano rice and spherical structures. The SEM and TEM images confirmed the shape variation. The PL spectra of the samples produced showed broad band profiles, with the highest emissions occurring in the blue and green regions of the visible electromagnetic spectrum at approximately 515 nm. This behavior was caused by the presence of bulk, surface, and interface defects, which resulted in a large density of intermediate energy levels (both shallow and deep) in the forbidden band. The results of Zeta potential analysis indicated a change in charge carriers caused by variations in pH during annealing at 600°C. Additionally, the results of PALS demonstrated that the HAp sample with pH11 annealed at 600°C had the highest oxygen vacancy defects. These defects create charge carriers, trapping centers, and emit light signals, contributing to an increase in the photoluminescence effect. Our findings indicate that the hexagonal HAp fabricated with pH11 annealed at 600°C is the most defective structure, which enhances the photoluminescence effect. Therefore, it can be an excellent material for phototherapy to track tumor cells.

## Data Availability

All data generated or analyzed during this study are included in this published article.

## References

[1] Sobczak-Kupiec, A. *et al.* Review of the applications of biomedical compositions containing hydroxyapatite and collagen modified by bioactive components. *Materials (Basel)* **14**, 2096. 10.3390/ma14092096 (2021). 33919199 10.3390/ma14092096 PMC8122483

[2] Machado, T. R. *et al.* Structural properties and self-activated photoluminescence emissions in hydroxyapatite with distinct particle shapes. *Ceram. Int.* **44**, 236–245. 10.1016/j.ceramint.2017.09.164 (2018).

[3] Uskoković, V. The role of hydroxyl channel in defining selected physicochemical peculiarities exhibited by hydroxyapatite. *RSC Adv.* **5**, 36614–36633. 10.1039/c4ra17180b (2015). 26229593 10.1039/C4RA17180B PMC4517856

[4] Diez-Escudero, A., Andersson, B., Persson, C. & Hailer, N. P. Hexagonal pore geometry and the presence of hydroxyapatite enhance deposition of mineralized bone matrix on additively manufactured polylactic acid scaffolds. *Mater. Sci. Eng. C* **125**, 112091. 10.1016/j.msec.2021.112091 (2021). 10.1016/j.msec.2021.112091 33965101

[5] Ratha, I., Datta, P., Balla, V. K., Nandi, S. K. & Kundu, B. Effect of doping in hydroxyapatite as coating material on biomedical implants by plasma spraying method: A review. *Ceram. Int.* **47**, 4426–4445. 10.1016/j.ceramint.2020.10.112 (2021).

[6] Mushtaq, A. *et al.* Magnetic hydroxyapatite nanocomposites: The advances from synthesis to biomedical applications. *Mater. Des.* **197**, 109269. 10.1016/j.matdes.2020.109269 (2021).

[7] Ibrahim, M. & Dawood, A. Influence of doping chromium ions on the electrical properties of hydroxyapatite. *Egypt. J. Basic Appl. Sci.* **7**, 35–46. 10.1080/2314808X.2019.1710055 (2020).

[8] Beh, C. Y. *et al.* Morphological and optical properties of porous hydroxyapatite/cornstarch (HAp/Cs) composites. *J. Mater. Res. Technol.* **9**, 14267–14282. 10.1016/j.jmrt.2020.10.012 (2020).

[9] Ma, G. Three common preparation methods of hydroxyapatite. *IOP Conf. Ser. Mater. Sci. Eng.* **688**, 033057. 10.1088/1757-899X/688/3/033057 (2019).

[10] Bokov, D. *et al.* Nanomaterial by Sol-Gel Method: Synthesis and application. *Adv. Mater. Sci. Eng.* **2021**, 1–21. 10.1155/2021/5102014 (2021).

[11] Ebrahimi, S., Mohd Nasri, C. S. S. & Bin Arshad, S. E. Hydrothermal synthesis of hydroxyapatite powders using Response Surface Methodology (RSM). *PLoS One* **16**, 1–24. 10.1371/journal.pone.0251009 (2021). 10.1371/journal.pone.0251009 PMC8136633 34014966

[12] Yelten-Yilmaz, A. & Yilmaz, S. Wet chemical precipitation synthesis of hydroxyapatite (HA) powders. *Ceram. Int.* **44**, 9703–9710. 10.1016/j.ceramint.2018.02.201 (2018).

[13] Mihara, A. *et al.* Photoluminescent layered crystal consisting of anderson-type polyoxometalate and surfactant toward a potential inorganic–Organic hybrid laser. *Int. J. Mol. Sci.* **25**, 345. 10.3390/ijms25010345 (2023). 38203515 10.3390/ijms25010345 PMC10778674

[14] Feng, A. & Smet, P. F. A review of mechanoluminescence in inorganic solids: Compounds, mechanisms, models and applications. *Materials (Basel)* **11**, 484. 10.3390/ma11040484 (2018). 29570650 10.3390/ma11040484 PMC5951330

[15] Kargozar, S. *et al.* Hydroxyapatite nanoparticles for improved cancer theranostics. *J. Funct. Biomater.* **13**, 100. 10.3390/jfb13030100 (2022). 35893468 10.3390/jfb13030100 PMC9326646

[16] Zantye, P., Fernandes, F., Ramanan, S. R. & Kowshik, M. Rare earth doped hydroxyapatite nanoparticles for in vitro bioimaging applications. *Curr. Phys. Chem.* **9**, 94–109. 10.2174/1877946809666190828104812 (2019).

[17] Lara-Ochoa, S., Ortega-Lara, W. & Guerrero-Beltrán, C. E. Hydroxyapatite nanoparticles in drug delivery: Physicochemistry and applications. *Pharmaceutics* **13**, 1–24. 10.3390/pharmaceutics13101642 (2021). 10.3390/pharmaceutics13101642 PMC8537309 34683935

[18] Wang, S. *et al.* In situ assembly of Mt-HAP drug carrier with pH-responsive sustained release properties. *Mater. Res. Express* **7**, 095006. 10.1088/2053-1591/abb630 (2020).

[19] Han, W., Chae, S. H., Kim, T., Lee, D. & Kim, H. White-light-emitting triphasic fibers as a phosphor for light-emitting diodes. *Nanoscale Adv.* **2**, 5403–5411. 10.1039/d0na00396d (2020). 36132022 10.1039/d0na00396d PMC9418951

[20] Tite, T. *et al.* Cationic substitutions in hydroxyapatite: Current status of the derived biofunctional effects and their in vitro interrogation methods. *Materials (Basel)* **11**, 1–62. 10.3390/ma11112081 (2018). 10.3390/ma11112081 PMC6266948 30355975

[21] Zhang, Z. *et al.* Synthesis of Tb 3+-doped Ca-deficient hydroxyapatite and its photoluminescence for white light-emitting diode application. *Adv. Mater. Res.* **560–561**, 825–829. 10.4028/www.scientific.net/AMR.560-561.825 (2012).

[22] Tesch, A. *et al.* Luminomagnetic Eu3 +- and Dy3 +-doped hydroxyapatite for multimodal imaging. *Mater. Sci. Eng. C* **81**, 422–431. 10.1016/j.msec.2017.08.032 (2017). 10.1016/j.msec.2017.08.032 28887994

[23] Neacsu, I. A., Stoica, A. E., Vasile, B. S. & Andronescu, E. Luminescent hydroxyapatite doped with rare earth elements for biomedical applications. *Nanomaterials* **9**, 239. 10.3390/nano9020239 (2019). 30744215 10.3390/nano9020239 PMC6409594

[24] Mondal, S. *et al.* Rare earth element doped hydroxyapatite luminescent bioceramics contrast agent for enhanced biomedical imaging and therapeutic applications. *Ceram. Int.* **46**, 29249–29260. 10.1016/j.ceramint.2020.08.099 (2020).

[25] Kolesnikov, I. E., Nikolaev, A. M., Lähderanta, E., Frank-Kamenetskaya, O. V. & Kuz’mina, M. A. Structural and luminescence properties of Ce3+-doped hydroxyapatite nanocrystalline powders. *Opt. Mater. (Amst.)* **99**, 109550. 10.1016/j.optmat.2019.109550 (2020).

[26] Machado, T. R. *et al.* A novel approach to obtain highly intense self-activated photoluminescence emissions in hydroxyapatite nanoparticles. *J. Solid State Chem.* **249**, 64–69. 10.1016/j.jssc.2016.12.018 (2017).

[27] Machado, T. R. *et al.* Designing biocompatible and multicolor fluorescent hydroxyapatite nanoparticles for cell-imaging applications. *Mater. Today Chem.* **14**, 100211. 10.1016/j.mtchem.2019.100211 (2019).

[28] Rodríguez-Lugo, V. *et al.* Wet chemical synthesis of nanocrystalline hydroxyapatite flakes: Effect of pH and sintering temperature on structural and morphological properties. *R. Soc. Open Sci.* **5**, 180962. 10.1098/rsos.180962 (2018). 30225084 10.1098/rsos.180962 PMC6124097

[29] Jyotsna, V. P. Synthesis and characterization of hydroxyapatite nanoparticles and their cytotoxic effect on a fish vertebra derived cell line. *Biocatal. Agric. Biotechnol.* **25**, 101612. 10.1016/j.bcab.2020.101612 (2020).

[30] Neves, J. G. *et al.* Effect of pH level and calcination on the production of calcium phosphates by acidic route of wet precipitation. *Ceramica* **67**, 236–43. 10.1590/0366-69132021673822965 (2021).

[31] Xidaki, D. *et al.* Synthesis of hydroxyapatite, β-Tricalcium phosphate and biphasic calcium phosphate particles to act as local delivery carriers of curcumin: Loading, release and in vitro studies. *Materials (Basel)* **11**, 595. 10.3390/ma11040595 (2018). 29649121 10.3390/ma11040595 PMC5951479

[32] Guerrero-Gironés, J. *et al.* Biocompatibility of a ha/β-tcp/c scaffoldas a pulp-capping agent for vital pulp treatment: An in vivo study in rat molars. *Int. J. Environ. Res. Public Health* **18**, 3936. 10.3390/ijerph18083936 (2021). 33918101 10.3390/ijerph18083936 PMC8068992

[34] Disha, S. A., Sahadat Hossain, M., Habib, M. L. & Ahmed, S. Calculation of crystallite sizes of pure and metals doped hydroxyapatite engaging Scherrer method, Halder-Wagner method, Williamson-Hall model, and size-strain plot. *Results Mater.* **21**, 100496. 10.1016/j.rinma.2023.100496 (2024).

[35] Rabiei, M. *et al.* Comparing methods for calculating nano crystal size of natural hydroxyapatite using X-ray diffraction. *Nanomaterials* **10**, 1–21. 10.3390/nano10091627 (2020). 10.3390/nano10091627 PMC7557758 32825139

[36] Yudin, A. *et al.* Microwave treatment and pH influence on hydroxyapatite morphology and structure. *J. Phys. Conf. Ser.* **1145**, 012003. 10.1088/1742-6596/1145/1/012003 (2019).

[37] Kamieniak, J., Kelly, P. J., Banks, C. E. & Doyle, A. M. Mechanical, pH and thermal stability of mesoporous hydroxyapatite. *J. Inorg. Organomet. Polym. Mater.* **28**, 84–91. 10.1007/s10904-017-0652-3 (2018).

[38] Mohd Pu’ad, N. A. S. *et al.* Synthesis method of hydroxyapatite: A review. *Mater. Today Proc.* **29**, 233–9. 10.1016/j.matpr.2020.05.536 (2019).

[39] Wang, P. *et al.* Effects of synthesis conditions on the morphology of hydroxyapatite nanoparticles produced by wet chemical process. *Powder Technol.* **203**, 315–321. 10.1016/j.powtec.2010.05.023 (2010).

[40] Dhand, V., Rhee, K. Y. & Park, S. J. The facile and low temperature synthesis of nanophase hydroxyapatite crystals using wet chemistry. *Mater. Sci. Eng. C* **36**, 152–159. 10.1016/j.msec.2013.11.049 (2014). 10.1016/j.msec.2013.11.049 24433898

[41] Miyajima, H., Touji, H. & Iijima, K. Hydroxyapatite particles from simulated body fluids with different ph and their effects on mesenchymal stem cells. *Nanomaterials* **11**, 1–11. 10.3390/nano11102517 (2021). 10.3390/nano11102517 PMC8538532 34684958

[42] Goh, K. W. *et al.* Effect of pH on the properties of eggshell-derived hydroxyapatite bioceramic synthesized by wet chemical method assisted by microwave irradiation. *Ceram. Int.* **47**, 8879–8887. 10.1016/j.ceramint.2020.12.009 (2021).

[43] Dizaj, S. M., Vazifehasl, Z., Salatin, S., Adibkia, K. & Javadzadeh, Y. Nanosizing of drugs: Effect on dissolution rate. *Res. Pharm. Sci.* **10**, 95–108 (2015). 26487886 PMC4584458

[44] Javadzadeh, Y. & Dizaj, S. M. *Recrystallization of Drugs—Effect on Dissolution Rate* (InTech, 2015).

[45] In, Y., Amornkitbamrung, U., Hong, M. H. & Shin, H. On the crystallization of hydroxyapatite under hydrothermal conditions: Role of sebacic acid as an additive. *ACS Omega* **5**, 27204–27210. 10.1021/acsomega.0c03297 (2020). 33134681 10.1021/acsomega.0c03297 PMC7594153

[46] Frost, R. L. *et al.* Raman spectroscopy of synthetic CaHPO4·2H2O- and in comparison with the cave mineral brushite. *J. Raman Spectrosc.* **43**, 571–576. 10.1002/jrs.3063 (2012).

[47] Solovyeva, E. V., Odintsova, O. V., Svinko, V. O., Makeeva, D. V. & Danilov, D. V. Hydroxyapatite-nanosilver composites with plasmonic properties for application in surface-enhanced Raman spectroscopy. *Mater. Today Commun.* **35**, 105908. 10.1016/j.mtcomm.2023.105908 (2023).

[48] Stammeier, J. A., Purgstaller, B., Hippler, D., Mavromatis, V. & Dietzel, M. In-situ Raman spectroscopy of amorphous calcium phosphate to crystalline hydroxyapatite transformation. *MethodsX* **5**, 1241–1250. 10.1016/j.mex.2018.09.015 (2018). 30364715 10.1016/j.mex.2018.09.015 PMC6197615

[49] Le Parc, R. *et al.* Infrared and Raman spectroscopy of non-conventional hydrogen bonding between: N, N ′-disubstituted urea and thiourea groups: A combined experimental and theoretical investigation. *Phys. Chem. Chem. Phys.* **21**, 3310–3317. 10.1039/c8cp06625f (2019). 30688324 10.1039/c8cp06625f

[50] Timchenko, P. E. *et al.* Experimental studies of hydroxyapatite by Raman spectroscopy. *J. Opt. Technol.* **85**, 130. 10.1364/jot.85.000130 (2018).

[51] Wang, Y., Moo, Y. X., Chen, C., Gunawan, P. & Xu, R. On the crystallization of hydroxyapatite under hydrothermal conditions: Role of sebacic acid as an additive. *J. Colloid Interface Sci.* **352**, 393–400. 10.1016/j.jcis.2010.08.060 (2010). 20846664 10.1016/j.jcis.2010.08.060

[52] Hadjiivanov, K. I. *et al.* Power of infrared and raman spectroscopies to characterize metal-organic frameworks and investigate their interaction with guest molecules. *Chem. Rev.* **121**, 1286–1424. 10.1021/acs.chemrev.0c00487 (2021). 33315388 10.1021/acs.chemrev.0c00487

[53] Bishop, J. L. *et al.* Spectral properties of anhydrous carbonates and nitrates. *Earth Sp. Sci.*10.1029/2021EA001844 (2021).

[54] Nandiyanto, A. B. D., Oktiani, R. & Ragadhita, R. How to read and interpret ftir spectroscope of organic material. *Indones. J. Sci. Technol.* **4**, 97–118. 10.17509/ijost.v4i1.15806 (2019).

[55] Goloshchapov, D., Seredin, P., Minakov, D. & Domashevskaya, E. Study of the nanoporous CHAP photoluminiscence for developing the precise methods of early caries detection. *IOP Conf. Ser. Mater. Sci. Eng.* **307**, 012027. 10.1088/1757-899X/307/1/012027 (2018).

[56] Van, H. N., Tam, P. D., Kien, N. D. T., Huy, P. T. & Pham, V. H. Enhancing the luminescence of Eu3+/Eu2+ ion-doped hydroxyapatite by fluoridation and thermal annealing. *Luminescence* **32**, 817–823. 10.1002/bio.3257 (2017). 28028916 10.1002/bio.3257

[57] Yang, L. *et al.* Structural responses of metallic glasses under neutron irradiation. *Sci. Rep.* **7**, 1–13. 10.1038/s41598-017-17099-2 (2017). 29196681 10.1038/s41598-017-17099-2 PMC5711955

[58] Dinh V-P, Luu AT, Krzysztof S, Kozlenko D, Khiem L, Dang NT, et al. Crystallization pathways, morphologies and structural defects of α-MnO2 nanomaterial synthesized under annealed temperatures (2020).

[59] Gomaa, E., Mazzroua, A. & Mohamed, M. Comparison between the effects of alcohols and diols on polymethyl-methacrylate and polyacrylamide with positron annihilation lifetime and electric conductivity measurements. *J. Appl. Polym. Sci.* **88**, 3078–3083 (2003).

[60] Mahmoud, K. R., Al-Sigeny, S., Sharshar, T. & El-Hamshary, H. Positron annihilation study on free volume of amino acid modified, starch-grafted acrylamide copolymer. *Radiat. Phys. Chem.* **75**, 590–595. 10.1016/j.radphyschem.2005.12.037 (2006).

[61] Elsayed, M., Staab, T. E. M., Čížek, J. & Krause-Rehberg, R. Monovacancy-hydrogen interaction in pure aluminum: Experimental and ab-initio theoretical positron annihilation study. *Acta Mater.* **248**, 118770. 10.1016/j.actamat.2023.118770 (2023).

[62] Taylor, C. N., Shimada, M., Watkins, J. M., Hu, X. & Oya, Y. Neutron irradiated tungsten bulk defect characterization by positron annihilation spectroscopy. *Nucl. Mater. Energy* **26**, 100936. 10.1016/j.nme.2021.100936 (2021).

[63] Biswas, D. *et al.* Structural defects characterization of silver-phosphate glass nanocomposites by positron annihilation and related experimental studies. *Mater. Charact.* **158**, 109928. 10.1016/j.matchar.2019.109928 (2019).

[64] Nishikawa, H. Thermal behavior of hydroxyapatite in structural and spectrophotometric characteristics. *Mater. Lett.* **50**, 364–370. 10.1016/S0167-577X(01)00318-4 (2001).

[65] Biswas, A., Das, S. K. & Sahoo, P. Oxidation issues during heat treatment and effect on the tribo-mechanical performance of electroless Ni-P–Cu deposits. *Proc. Inst. Mech. Eng. L J. Mater. Des. Appl.* **235**, 1665–85. 10.1177/1464420721999823 (2021).

[66] Cui, W. *et al.* Adsorption behaviors of different water structures on the fluorapatite (001) surface: A DFT study. *Front. Mater.* **7**, 1–8. 10.3389/fmats.2020.00047 (2020).

[67] Okamoto, S. & Ohya-Nishiguchi, H. Fading mechanism of paint films composed of insoluble Azo pigment and titanium dioxide. *Bull. Chem. Soc. Japan* **63**, 2346–2351 (1990).

[68] Konstantinova, T. E., Ragulya, A. V., Doroshkevich, A. S., Volkova, G. K. & Glazunova, V. A. The mechanisms of particle formation in Y-doped ZrO 2 ’. *Int. J. Nanotechnol.* **3**, 29 (2006).

[69] Alkorta, I., Elguero, J. & Del Bene, J. E. Perturbing the O-H…O hydrogen bond in 1-OXO-3-hydroxy-2-propene. *Molecules* **26**, 1–12. 10.3390/molecules26113086 (2021). 10.3390/molecules26113086 PMC8196739 34064185

[70] Huerta, V. J., Fernández, P., Gómez, V., Graeve, O. A. & Herrera, M. Defect-related luminescence properties of hydroxyapatite nanobelts. *Appl. Mater. Today* **21**, 100822. 10.1016/j.apmt.2020.100822 (2020).

[71] Mahmoud, K. R., El-Shehawy, A. & Atta, H. Positron annihilation spectroscopy of chain end functionalized polystyrenes with definite numbers of benzyl alcohol and perfluorooctyl groups. *Polimeros*10.1590/0104-1428.04619 (2019).

[72] Afshar A, Ghorbani M, Ehsani N, Saeri MR, Sorrell CC. A study of zeta potential of plasma sprayed hydroxyapatite coating in four simulated physiological solutions. 16. (2003).

[73] Tollini, F. *et al.* Influence of pH on the kinetics of hydrolysis reactions: The case of epichlorohydrin and glycidol. *React. Chem. Eng.* **7**, 2211–2223. 10.1039/d2re00191h (2022).

[74] Barth, A. P., Tormena, C. F. & Viotto, W. H. pH influences hydrolysis of sodium polyphosphate in dairy matrices and the structure of processed cheese. *J. Dairy Sci.* **100**, 8735–8743. 10.3168/jds.2017-12764 (2017). 28843685 10.3168/jds.2017-12764

[75] Marinho, J. P. N. *et al.* Nanostructured system based on hydroxyapatite and curcumin: A promising candidate for osteosarcoma therapy. *Ceram. Int.* **49**, 19932–49. 10.1016/j.ceramint.2023.03.115 (2023).

[76] Kadu, K., Kowshik, M. & Ramanan, S. R. Tailoring of hydroxyapatite nanoparticle surfaces of varying morphologies to facilitate counterion diffusion and subsequent protein denaturation. *Biophys. Chem.* **296**, 106979. 10.1016/j.bpc.2023.106979 (2023). 36863072 10.1016/j.bpc.2023.106979

[77] Mahmoud, R., Mohamed, H. F. M., Hafez, S. H. M., Gadelhak, Y. M. & Abdel-Hady, E. E. Valorization of spent double substituted Co–Ni–Zn–Fe LDH wastewater nanoadsorbent as methanol electro-oxidation catalyst. *Sci. Rep.*10.1038/s41598-022-23798-2 (2022). 36369455 10.1038/s41598-022-23798-2 PMC9652425

[78] Ahmed, Y. M. Z., El-Sheikh, S. M. & Zaki, Z. I. Changes in hydroxyapatite powder properties via heat treatment. *Bull. Mater. Sci.* **38**, 1807–1819 (2015).

[79] Lošdorfer Božič, A. & Podgornik, R. pH dependence of charge multipole moments in proteins. *Biophys. J.* **113**, 1454–1465. 10.1016/j.bpj.2017.08.017 (2017). 28978439 10.1016/j.bpj.2017.08.017 PMC5627345

[80] Zampieron, C. I., Cesca, K., Faita, F. L., Immich, A. P. S. & Parize, A. L. Effect of heat-treatment temperature on the structure of calcium phosphate synthesized by wet precipitation. *Ceram. Int.*10.1016/j.ceramint.2023.06.204 (2023).

[81] Naqshbandi, A. & Rahman, A. Synthesis and characterization of chlorinated hydroxyapatite as novel synthetic bone substitute with negative zeta potential. *Ceram. Int.* **48**, 8112–8117. 10.1016/j.ceramint.2021.12.013 (2022).

