# Author Correction: Correlation between positron annihilation lifetime and photoluminescence measurements for calcined Hydroxyapatite

**DOI:** 10.1038/s41598-025-07154-8

**Published:** 2025-07-09

**Authors:** Hoda Atta, Kamal R. Mahmoud, Elsayed I. Salim, Eithar Elmohsnawy, Abdelhamid El‑Shaer

**Affiliations:** 1 Physics Department, Faculty of Science, Kafrelsheikh University, Kafrelsheikh, 33516 Egypt; 2 Research Lab. of Molecular Carcinogenesis, Zoology Department, Faculty of Science, Tanta University, Tanta, 31527 Egypt; 3 Department of Botany, Faculty of Science, Kafrelsheikh University, Kafrelsheikh, 33516 Egypt

Correction to: *Scientific Reports* 10.1038/s41598-024-59855-1, published online 06 May 2024

The original version of this Article contained an error in the spelling of the author Elsayed I Salim which was incorrectly given as El Sayed I Salim.

The original Article has been corrected.

